# Supplementary material for: Modeling glioblastoma heterogeneity as a dynamic network of cell states
Source: Mol Syst Biol. 2021 Sep 16;17(9):e10105. doi: 10.15252/msb.202010105 (PMC8444284; doi:10.15252/msb.202010105)
Supplement: Supplementary file 6 — Source Data for Figure 5 [file MSB-17-e10105-s004.zip › Figure5A_sourcedata/GSEA_3017/nosigdb_stateD.GseaPreranked.1622795010397/RICHARDS_NOWAKOWSKI_SCIENCE_2017_NIN4_UPREG.html]

Details for gene set RICHARDS\_NOWAKOWSKI\_SCIENCE\_2017\_NIN4\_UPREG[GSEA]

|  || Dataset | state23017 |
| Phenotype | NoPhenotypeAvailable |
| Upregulated in class | na\_pos |
| GeneSet | RICHARDS\_NOWAKOWSKI\_SCIENCE\_2017\_NIN4\_UPREG |
| Enrichment Score (ES) | 0.19257456 |
| Normalized Enrichment Score (NES) | 1.2411221 |
| Nominal p-value | 0.21359223 |
| FDR q-value | 0.350154 |
| FWER p-Value | 0.818 |
Table: GSEA Results Summary

  

Fig 1: Enrichment plot: RICHARDS\_NOWAKOWSKI\_SCIENCE\_2017\_NIN4\_UPREG      
 Profile of the Running ES Score & Positions of GeneSet Members on the Rank Ordered List

  

| PROBE | GENE SYMBOL | GENE\_TITLE | RANK IN GENE LIST | RANK METRIC SCORE | RUNNING ES | CORE ENRICHMENT || 1 | SOX11 |  |  | 6 | 0.578 | 0.0535 | Yes |
| 2 | RAB13 |  |  | 43 | 0.367 | 0.0626 | Yes |
| 3 | RPL10 |  |  | 53 | 0.344 | 0.0902 | Yes |
| 4 | RPS23 |  |  | 78 | 0.313 | 0.1032 | Yes |
| 5 | SOX4 |  |  | 91 | 0.298 | 0.1239 | Yes |
| 6 | MARCKS |  |  | 96 | 0.295 | 0.1504 | Yes |
| 7 | RPS11 |  |  | 113 | 0.279 | 0.1661 | Yes |
| 8 | CHD7 |  |  | 116 | 0.278 | 0.1926 | Yes |
| 9 | PLXNA4 |  |  | 182 | -0.252 | 0.1677 | No |
| 10 | FTL |  |  | 271 | -0.261 | 0.1258 | No |
| 11 | BBX |  |  | 368 | -0.270 | 0.0788 | No |
| 12 | INTS10 |  |  | 409 | -0.274 | 0.0754 | No |
| 13 | WLS |  |  | 456 | -0.280 | 0.0680 | No |
| 14 | DLX1 |  |  | 490 | -0.284 | 0.0710 | No |
| 15 | FBXW7 |  |  | 494 | -0.284 | 0.0973 | No |
| 16 | SNTG1 |  |  | 697 | -0.319 | -0.0269 | No |
| 17 | TMSB4X |  |  | 742 | -0.329 | -0.0278 | No |
| 18 | TMEM245 |  |  | 788 | -0.340 | -0.0284 | No |
| 19 | NDFIP1 |  |  | 805 | -0.346 | -0.0060 | No |
| 20 | PCM1 |  |  | 834 | -0.353 | 0.0079 | No |
| 21 | RND3 |  |  | 853 | -0.358 | 0.0300 | No |
| 22 | MEG3 |  |  | 975 | -0.404 | -0.0229 | No |
| 23 | CEP78 |  |  | 984 | -0.408 | 0.0119 | No |
| 24 | DCLK2 |  |  | 986 | -0.408 | 0.0521 | No |
| 25 | KIFAP3 |  |  | 988 | -0.409 | 0.0925 | No |
| 26 | AKAP12 |  |  | 994 | -0.412 | 0.1301 | No |
| 27 | CPEB4 |  |  | 1090 | -0.465 | 0.1034 | No |
| 28 | PBX1 |  |  | 1261 | -0.733 | 0.0456 | No |
Table: GSEA details [plain text format]

  

Fig 2: RICHARDS\_NOWAKOWSKI\_SCIENCE\_2017\_NIN4\_UPREG: Random ES distribution      
 Gene set null distribution of ES for **RICHARDS\_NOWAKOWSKI\_SCIENCE\_2017\_NIN4\_UPREG**
